# Chemical Structure of EVA Films Obtained by Pulsed Electron Beam and Pulse Laser Ablation

**DOI:** 10.3390/polym11091419

**Published:** 2019-08-29

**Authors:** Agata Niemczyk, Dariusz Moszyński, Roman Jędrzejewski, Konrad Kwiatkowski, Joanna Piwowarczyk, Jolanta Baranowska

**Affiliations:** 1 Faculty of Mechanical Engineering and Mechatronics, Institute of Materials Science and Engineering, West Pomeranian University of Technology, Szczecin, al. Piastow 19, 70-310 Szczecin, Poland; 2 Faculty of Chemical Technology and Engineering, Institute of Inorganic Chemical Technology and Environment Engineering, West Pomeranian University of Technology, Szczecin, al. Piastow 42, 71-065 Szczecin, Poland; 3 Łukasiewicz Research Network—PORT Polish Center for Technology Development, ul. Stabłowicka 147, 54-066 Wrocław, Poland; 4 Faculty of Mechanical Engineering and Mechatronics, Department of Mechanics and Fundamentals of Machine Design, West Pomeranian University of Technology, Szczecin, al. Piastow 19, 70-310 Szczecin, Poland

**Keywords:** poly(ethylene-*co*-vinyl acetate), pulsed electron beam deposition, pulsed laser deposition, chemical structure analysis

## Abstract

Poly(ethylene-*co*-vinyl acetate) (EVA) films were deposited for the first time using physical methods. The chemical structure of the films obtained using two techniques, pulsed electron beam deposition (PED) and pulsed laser deposition (PLD), was studied by attenuated total reflection Fourier infrared spectroscopy (ATR-FTIR) and X-ray photoelectron spectroscopy (XPS). Whilst significant molecular degradation of the EVA films was observed for the PLD method, the original macromolecular structure was only partially degraded when the PED technique was used, emphasizing the superiority of the PED method over PLD for structurally complex polymers such as EVA. Optical and scanning electron microscopic observations revealed compact and smooth EVA films deposited by pulsed electron beam ablation as opposed to heterogeneous films with many different sized particulates obtained by PLD.

## 1. Introduction

Physical vapor deposition (PVD) is an important approach in polymer thin film development. Although it is generally accepted that using any PVD method is a good way to obtain a polymeric film with different tunable properties, they still have considerable limitations due to the complex nature of the macromolecules [1]. Key challenges that need to be overcome are material degradation and chemical structure change, which are particularly problematic for polymers with high molecular weight [2]. The observed chemical alteration of the polymer chain is mainly attributed to the mechanism of the PVD process. For the majority of PVD techniques, transformation of the material (target) into vapor is based on thermal effects and then evaporation or sublimation. However, considering the size of macromolecules and the complexity of their molecular intra- and interactions, maintaining structural intactness under such conditions is highly unfavorable thermodynamically, leading to the chain scission [3].

A breakthrough in the development of PVD polymer films was the successful laser ablation (pulsed laser deposition technique, PLD) of polytetrafluoroethylene with thin film formation, which had quite similar structure to the target material [4,5]. This contributed to an increase in the intensity of further research as well as to the parallel development of the pulsed electron beam deposition method (PED) [6,7,8]. The PED technique seems to be an interesting alternative for polymer film deposition, being independent of the optical properties of the material. This is because electrons can interact via Coulomb interactions with any material, both inorganic and organic, transferring energy into the target surface more effectively than photons (as in PLD) [9]. The first results in polymer thin films development using the PED technique indicated a greater potential of this method (vs PLD), leading to the conclusion that the films obtained can have almost the same stoichiometry of the polymer structure as the target [6,7,8,10].

Nevertheless, the possibility of obtaining a polymer film with exactly the same structure as the target opens up a question regarding the mechanism of this process. Hitherto the research has focused on polymers characterized by simple chemical structure (without functional groups) such as some polyolefins and fluoropolymers, which even in the case of significant chain scission could re-polymerize to a structure similar to the target material (similar to the PLD mechanism) [4]. The application of the PED technique to produce thin films of more structurally diverse polymers, e.g., copolymers, remains an open issue. What is more, detailed chemical structure assessment of the film obtained is also needed for improved understanding of the underlying processes involved with this method.

Poly(ethylene-*co*-vinyl acetate) (EVA) is a widely used polymer for diverse applications, combining advantages of polyethylene (Et) and poly(vinyl acetate) (VAc) such as flexibility, toughness (in a broad range of temperatures) and stress crack resistance [11,12,13]. Good adhesion to a wide range of materials (such as metals, ceramics and polymers) is a main advantage of EVA (and its blends) coatings, and thus an important reason why they are extensively used in diverse applications. Among the many worth mentioning are drug releasing [14], heat-sealable [15], or anticorrosive [16], as well as coatings and adhesives for solar and optical fiber application [12,17]. Nonetheless, most commonly used methods of the coating fabrication such as spray technique or wet laminating require dissolving the coating material in some organic solvent, and what is more lead to thick layers, in which thicknesses range from several dozen to several hundred microns.

**Figure 1:**
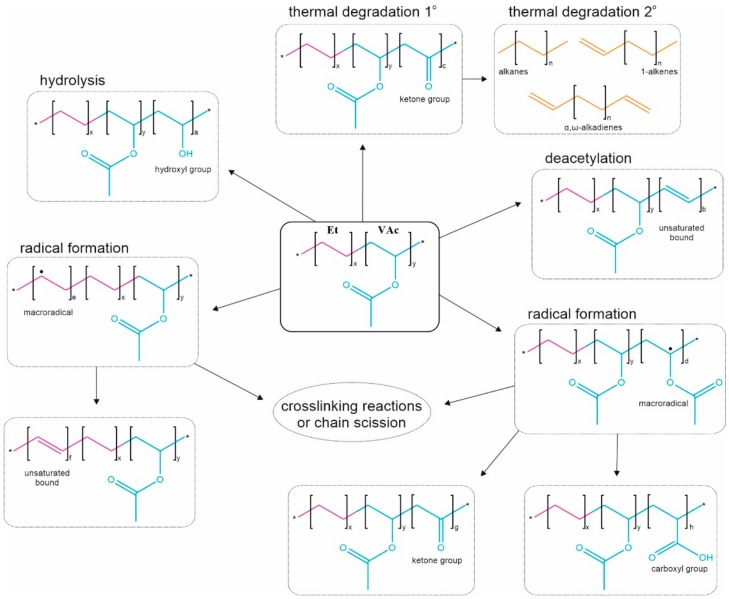
Possible chemical structure alterations of poly(ethylene-*co*-vinyl acetate) (EVA) copolymer.

Due to the industrial importance of EVA, much research has been carried out. The impact of various physical factors such as temperature, visible light, UV, electron beam and gamma radiation on the chemical structure of the copolymer has been determined in detail, referring especially to its crosslinking degree and degradation [18,19,20,21,22,23]. The most common structural change that the EVA macromolecules undergo is the formation of ketone (or aldehyde) groups, mostly as a result of the thermal elimination of acetaldehyde or acetic acid (from VAc unit) [22,24]. The thermal degradation process can continue, resulting in the formation of free radicals (from both VAc and Et units). Free radicals can also be created as a consequence of UV, electron or γ-irradiation [20,22]. A further consequence of the presence of free radicals in the main chain is a disproportional termination followed by chain unsaturation, crosslinking and scission of macromolecules [25]. Hydrolysis of the ester group (VAc unit) with the formation of vinyl alcohol (VA) occurs less frequently (if it is not performed deliberately), and leads to the Et-VAc-VA terpolymer, characterized by improved barrier properties [26]. Other structural changes that occur rarely are deacetylation and hydrolysis (VAc) leading, respectively, to the formation of an unsaturated bond and a carboxyl group [21]. The typical ways of EVA chemical structure modification are presented in Figure 1.

The aim of our work was to evaluate the chemical structure of thin films of EVA copolymer obtained for the first time by two different PVD methods, i.e., pulsed laser and pulsed electron beam deposition. Using well-established knowledge regarding neat and physically treated EVA, we have attempted to describe the changes induced by photons or electrons in the chemical structure of the macromolecules in the deposited films. We believe that the study of more structurally-diverse polymers such as the poly(ethylene-*co*-vinyl acetate) copolymer can lead to a deeper understanding of the ongoing processes and of the macromolecular changes occurring during the ablation and film formation, revealing its complexity and the need for further in-depth investigation.

The secondary objective of the presented work was to verify the usefulness of the PED and PLD techniques in EVA thin film development, which is important considering the high potential for application of these films, especially in the crosslinked state [27].

## 2. Materials and Methods

### 2.1. Film Preparation

EVA copolymer coatings were deposited by means of a PED/PLD system (NEOCERA, Inc., Beltsville, MD, USA). ATEVA4030AC copolymer (Celanese Corporation, Irving, TX, USA) was used with 40 wt% of vinyl acetate. The target material (the disc of 50 mm diameter and 10 mm height) was prepared by injection molding according to the producer’s Datasheet. The EVA coatings were deposited on silicon (100) substrates 10 × 10 mm in size. Prior to film deposition, the substrates were sonically cleaned in an acetone bath, rinsed in acetone and isopropyl alcohol and dried in an air flow. In the case of the PED method, a pulse electron beam source (PEBS) was used, operating at a voltage of 12 kV, which corresponded to a pulse energy of 200 mJ. The pulse width was 100 ns. In the case of the PLD method, an excimer laser (Coherent CompexPro 201F; He/Ne;KrF, λ = 248 nm, Santa Clara, CA, USA) with 20 ns pulse duration was used. The pulse energy was set to 80 mJ. For both techniques, the following parameters were kept constant: 5 Hz pulse repetition rate; 80 mm distance between the target and substrate; nitrogen as the background gas at a pressure of 0.93 Pa; deposition time corresponding to 25,000 pulses.

### 2.2. Morphology Study

The surface morphology of the films was evaluated by light microscopy (LM) observation using a Nikon MM-40 microscope (Nikon, Tokyo, Japan), and by scanning electron microscopy (SEM) using a Hitachi SU-70 microscope (Hitachi, Tokyo, Japan). SEM secondary electron images (SEI) were obtained at an accelerating voltage of 5 kV.

### 2.3. Structure Determination

The chemical structures of EVA films were characterized using attenuated total reflection Fourier infrared spectroscopy (ATR-FTIR; Lumos, Bruker, Billerica, MA, USA). Sixty four scans at a resolution of 4 cm^−1^ were carried out for each sample. Each spectrum was collected with an air background and was corrected for CO_2_ and H_2_O. All spectra are presented after baseline correction and in the wave number range of 4000–600 cm^−1^.

The X-ray photoelectron spectra were obtained using Al *Kα* (hν = 1486.6 eV) radiation with a Prevac (Rogów, Poland) system equipped with a Scienta SES 2002 electron energy analyzer operating at constant transmission energy (E_p_ = 50 eV). The spectrometer was calibrated by using the following photoemission lines (with reference to the Fermi level): EB Cu 2p_3/2_ = 932.8 eV, EB Ag 3d_5/2_ = 368.3 eV. The instrumental resolution, as evaluated by the full-width at half maximum (FWHM) of the Ag 3d_5/2_ peak, was 1.0 eV. The samples were glued to a molybdenum sample holder. The analysis chamber during experiments was evacuated to a vacuum level better than 1 × 10^−9^ mbar. Data processing involved background subtraction by means of an “S-type” integral profile and a curve-fitting procedure (a mixed Gaussian–Lorentzian function was employed) based on a least-squares method (software CasaXPS). The experimental errors were estimated to be ±0.1 eV for the photoelectron peaks of carbon and oxygen. Charging effects were corrected by using the C 1s component ascribed after deconvolution to the aliphatic carbon bindings (component C–C), and set to 285.0 eV. The reproducibility of the peak position thus obtained was ±0.1 eV. The surface composition of the samples was calculated on the basis of the peak area intensities of the C 1s and O 1s transitions using the sensitivity factor approach and assuming homogeneous distribution of elements in the surface layer.

## 3. Results and Discussion

### 3.1. Film Morphology

**Figure 2:**
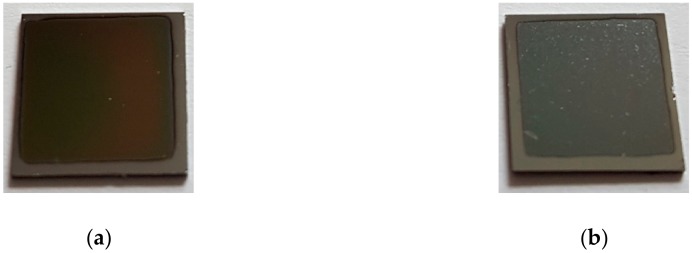
Macroscopic images of EVA_E (**a**) and EVA_L (**b**) films deposited on silicon wafer substrates.

The general appearance of the coatings obtained is shown in Figure 2. The coatings obtained in both processes covered the entire exposed surface. The visible uncovered rim is the result of the substrate being obscured by the holder during deposition. The film deposited by the PED technique (EVA_E) was transparent with a light brown color (Figure 2a), whereas obtained by PLD (EVA_L) was opaque and dark/black in color with strong non-uniformity of the surface (Figure 2b).

**Figure 3:**
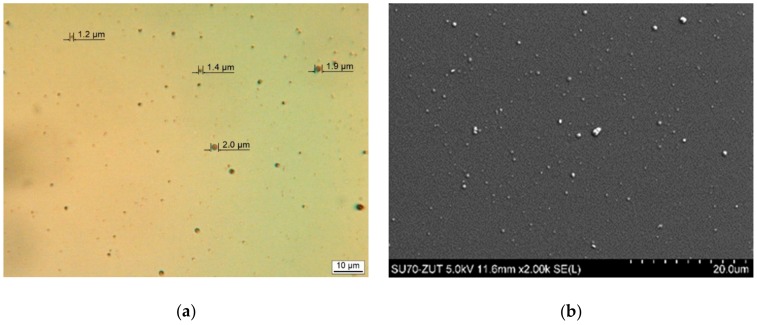
Microscopic pictures of EVA_E films deposited on silicon wafer substrate: (**a**) light (LM) and, (**b**) scanning electron microscopy (SEM, SEI).

**Figure 4:**
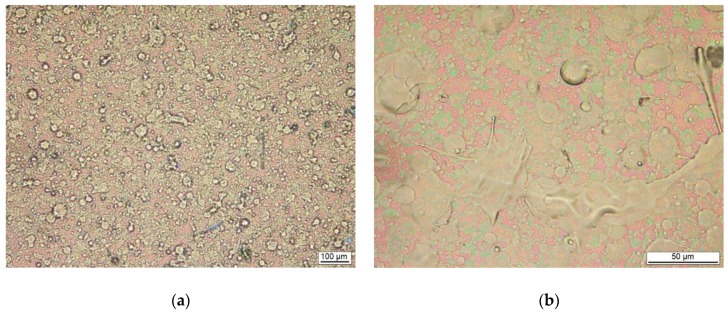

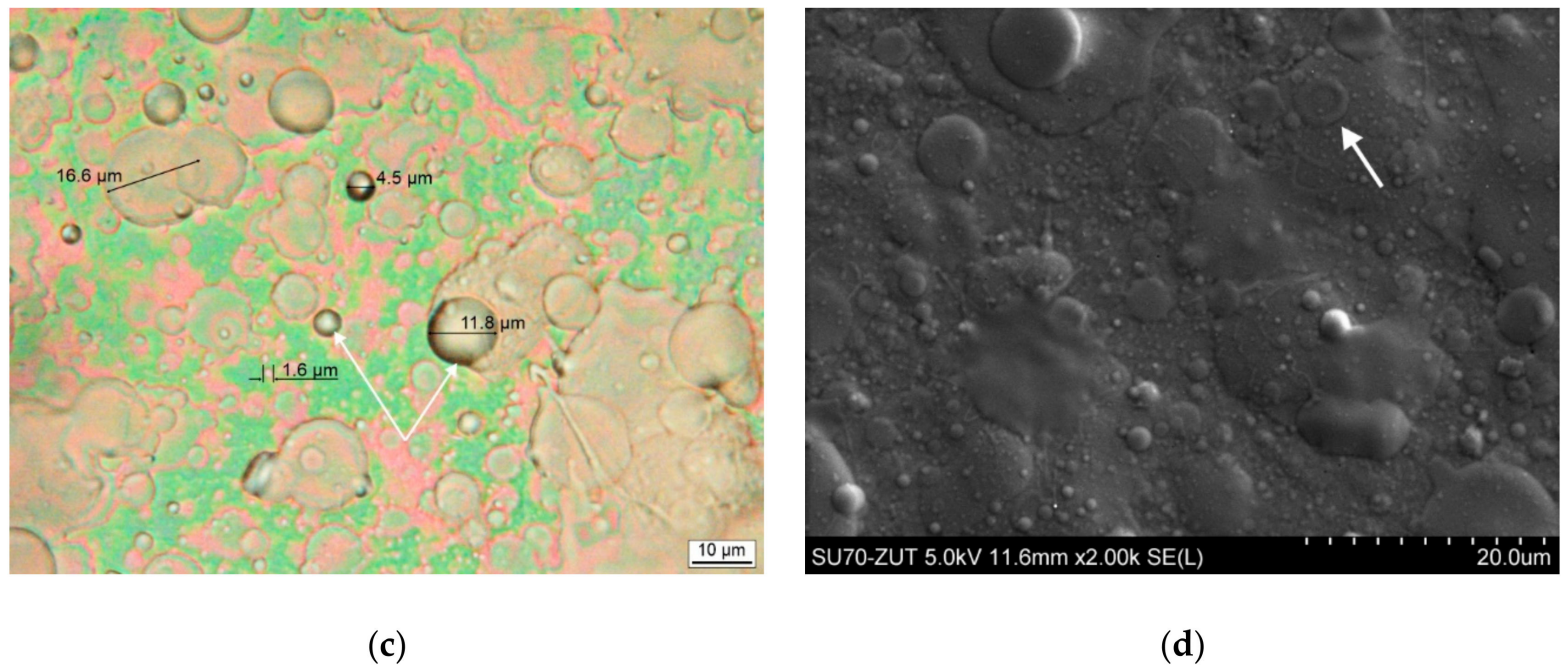
Microscopic pictures of EVA_L films deposited on silicon wafer substrate: (**a**–**c**) light (LM) and, (**d**) scanning electron microscopy (SEM, SEI). Description of the arrows is in the text.

Detailed analysis of the surfaces of both types of coatings demonstrated extreme differences in their morphology. EVA_E films were compact and smooth, as in Figure 3. Only small spherical particulates—called droplets—which are characteristic for the deposition of thin coatings by physical methods, could be seen on the surface. The size of these droplets did not exceed 2 micrometers (Figure 3a). Coatings obtained by the PLD method were very heterogeneous over the entire surface, as in Figure 4a. Examination of the surface at higher magnification by both light microscopy (Figure 4b and c) and scanning microscopy (Figure 4d) showed that the coating contained large particles of a partly spherical nature but also irregular “splash-like” shapes (Figure 4b). Spherical particles were also observed with diameters similar to those measured for EVA_E coatings (Figure 4c). However, most of the particles were larger—by the order of several micrometers (Figure 4c). Some of the spherical forms may be the remains of bubbles filled with gas at the deposition stage. These are indicated by the arrows in Figure 4c,d. Nevertheless, the surface of the coatings, especially because of the “splash” shapes, gives the impression of a type of spraying with liquid particles.

### 3.2. Chemical Structure

**Figure 5:**
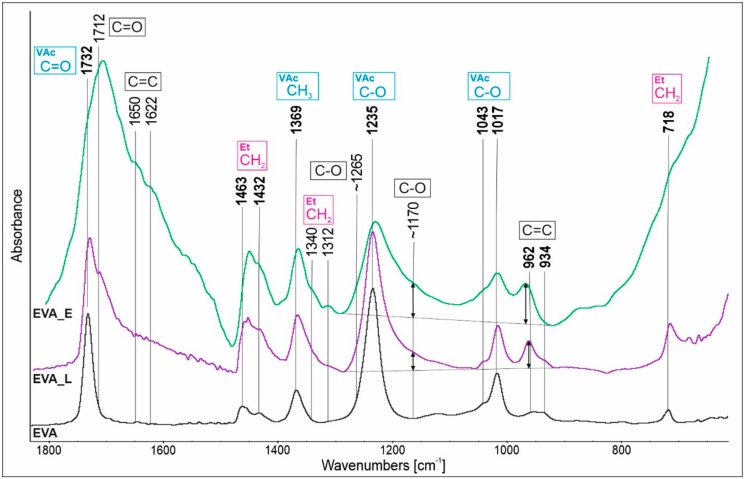
Fourier transform infrared spectroscopy (FTIR) spectra of EVA, EVA_L and EVA_D in the 1800–600 cm^−1^ region.

FTIR spectra of neat EVA (target material) and the films obtained by the PED and PLD methods are shown in Figure 5 in the wavenumber range of 1800–600 cm^−1^. EVA copolymer is characterized by typical absorption bands corresponding to the vinyl acetate block (1732 cm^−1^, 1369 cm^−1^, 1235cm^−1^, 1043 cm^−1^, 1017 cm^−1^) and ethylene block (1463 cm^−1^, 1432 cm^−1^, 718 cm^−1^) marked in bold and assigned to corresponding groups.

The differences between the spectra are evident, which indicates that the polymer structure has been changed during the laser and electron ablation. The differences are observed mainly in the regions of 1800–1500 cm^−1^ and 1300–1100 cm^−1^, and are more pronounced for EVA_E than for EVA_L. The absorption bands at 1732 cm^−1^ and 1235 cm^−1^, which corresponds to the C=O or C–O from the ester of the VAc block, have been broadened as a result of overlap with new bands originating mainly from ketone (1712 cm^−1^, 1170 cm^−1^) but also carboxylic acid (1712 cm^−1^, 1265 cm^−1^) functionalities.

The formation of the ketone group is recognized as a characteristic first step of the EVA copolymer degradation among which some low molecular weight by-products (acetaldehyde/acetic acid) are released. The formation of the ketone groups by the labile α-hydrogen elimination is a consequence of the strong inductive effect of the ester group (in VAc block) [22]. It has been reported after treatment of EVA film by an electron beam [28], γ-irradiation [21] or very high temperature [23]. Additionally, bands found in the 1800–1500 cm^−1^ absorption region at around 1650 and 1622 cm^−1^ are assigned to chain unsaturation which is confirmed by the occurrence of related bands as 962 and 934 cm^−1^. Interestingly, in comparing the absorbance of the ketone (1170 cm^−1^) and C=C (962 cm^−1^) bands, it can be noticed that for EVA_E the bands ratio is close to 1:1 and for EVA_L the unsaturation is 1:2.

Taking into account that evaporation/sublimation of macromolecules is thermodynamically limited, and that the molecular weight cannot exceed some specific value (for PTFE calculated to about 1000 Da according to [3]), we assume that the abovementioned structural changes i.e., VAc transformation to ketone and chain unsaturation take place most likely on the target surface at the beginning of the ablation process, probably in parallel with the main C–C chain scission (as the presence of C=C groups in the EVA copolymer indirectly indicates it [23]).

Additional chemical composition study of the surfaces of neat EVA, EVA_E and EVA_L films was performed using X-ray Photoelectron Spectroscopy. Only carbon and oxygen atoms were identified on the surface of all samples. The elemental composition of the surface calculated using the XPS data is given in Table 1. The elemental composition of the reference EVA sample corresponds to 35,2 wt% of the VAc block, which is slightly less than the value stated by the manufacturer (40 wt%), however it is recognized as an ethylene block enrichment of the surface typical for this copolymer. [13]. A limited presence of the adventitious carbon has to be considered as well.

**Figure 6:**
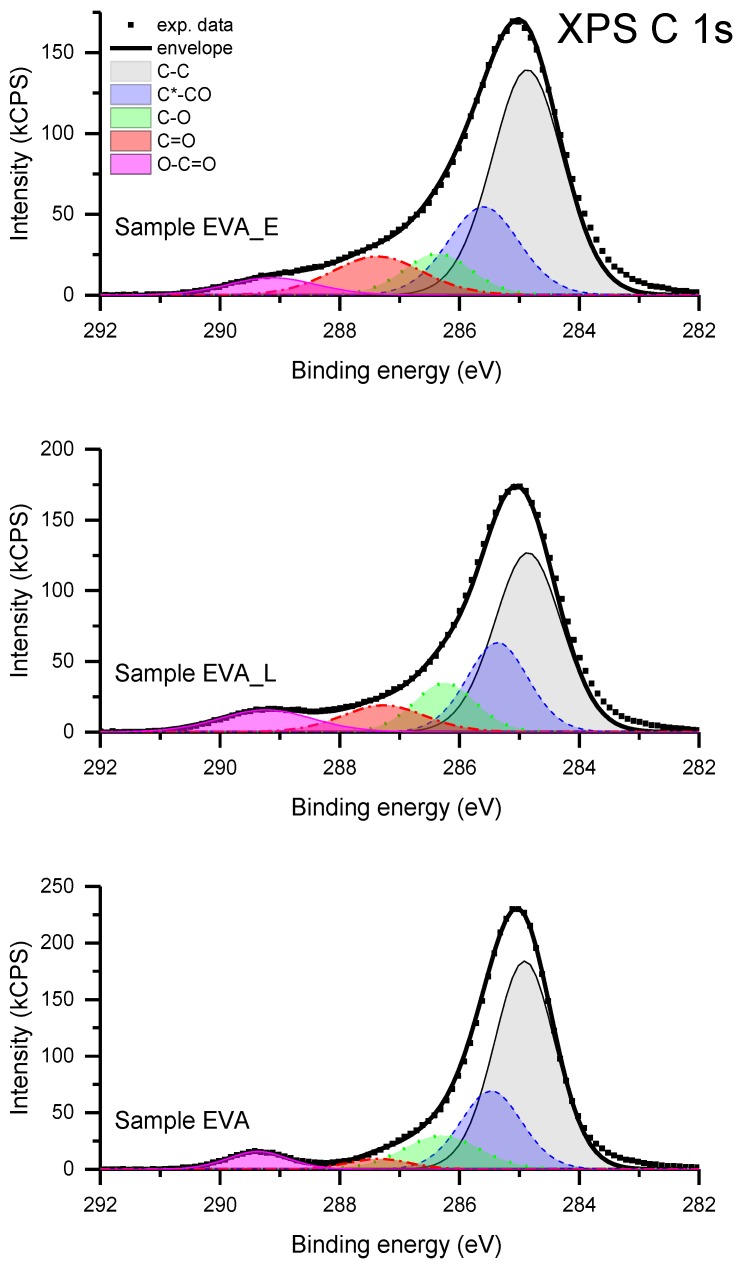
X-ray photoelectron spectroscopy (XPS) C 1s spectra for EVA, EVA_L and EVA_E films.

The detailed chemical composition evaluation of the examined materials is based on the analysis of the XPS C 1s peak. The spectra have a relatively complex profile (Figure 6). Deconvolution of the experimental data was carried out by applying a model consisting of five basic components of the C 1s transition. The component denoted as C–C, having a binding energy of 285.0 eV, is attributed to all non-functionalized carbon atoms, bonded either with a second carbon atom or with hydrogen atoms. The component denoted as C*–CO, shifted 0.5 ± 0.1 eV from component C–C in the direction of increasing binding energy, is ascribed to carbon atoms in the vicinity of oxygen atoms but not bound directly. The third component denoted as C–O, shifted 1.3±0.1 eV from component C–C in the direction of increasing binding energy, is ascribed to a group of differently-bonded carbon atoms linked to one atom of oxygen. The group comprises the functional groups C–O–C and C–OH, presumably present on the surface of the studied materials. The component C=O, shifted 2.4 ± 0.1 eV from component C–C in the direction of increasing binding energy, also corresponds to a set of functional groups: C=O, O–C–O. The last component, O–C=O, shifted 4.2 ± 0.1 eV from component C–C in the direction of increasing binding energy, is ascribed to a group of carbon atoms linked to more than one atom of oxygen. The binding energy assignments described above are based on the energy shifts given in Appendix E of [29]. The numerical deconvolution of the spectra resulted in the relative concentration of the surface functional group, which is given in Table 1. The total C 1s peak intensity is taken as 100.

The component C–C dominates in all materials due to a high content of Et blocks, however the elemental O/C ratio has been changed in the deposited films, indicating an increased amount of oxygen. To understand the influence of PLD and PED deposition methods on the structural transformations of EVA, a relation between C=O and O–C=O components seems to be crucial. In the reference material the concentration of these functional groups is very small (the ratio is 0.6:1). After deposition by the PLD method the ratio between the C=O and O–C=O components is 1.1:1, while after deposition by PED it is 2.4:1. The amount of the C=O component, recognized by the FTIR spectra analysis as ketone groups, is almost two (for EVA_L) and four (for EVA_E) times greater than for neat material. This illustrates the scale of the first step of degradation that took place during the PLD/PED processes. Furthermore, the difference in the components ratio of the non-functionalized and oxygen bounded carbon atoms revealed an important difference between EVA_E and EVA_L films. Both deposited films are characterized by a lower C–C content, which suggests a decreased fraction of ethylene block in the final structure. Such a decrease may indicate the second step of the degradation, involving the degradation of the main chain and formation of volatile α,β-alkadienes, 1-alkens and alkanes [22,23]. The fact that the EVA_L material has fewer C=O groups but also less C–C component may indicate that, during the PLD process, the EVA macromolecules undergo structural destruction at a higher level than during electron ablation. This assumption is consistent with the IR analysis in which a lower amount of the ketone groups was also determined for EVA_L simultaneously with a higher C=C content, indicating the stronger chain scission thus further macromolecular degradation. This could also explain the observed significant difference in the morphology of the deposited films. In studies of the stability (aging) of EVA coatings, discoloration is a useful indicator for determining the degree of degradation: the darker the color, the greater the degree of degradation. A dark/black color of EVA_L films observed in our studies (Figure 2b) is described in the literature as “highly degraded” [23]. Moreover, as explained previously, the structural changes observed are associated with the release of volatile gaseous products of decomposition, which could justify the presence of bubble-like forms especially in the highly degraded EVA_L coatings (Figure 4d). It can also be hypothesized that in the case of the latter coatings, progressive degradation (and thus main chain scission) can explain the presence of the numerous droplets and splash-like particulates within the film (Figure 4). The molecules with lower molecular mass undergo more intensive thermal transition and therefore a kind of spraying can occur during the laser ablation.

It is proposed that the mechanism of polymer film creation by PVD techniques involves significant chain scission related to depolymerization, which in the case of structurally complex polymers, such as copolymers or polymers with heteroatoms in the structure, cannot take place and leads to monomer formation that is able to repolymerize by radical reaction on the substrate surface. Furthermore, the use of a radiation fluence that exceeds the dissociation energy of all bonds present in the polymer structure may lead to such intensive and random chain scission that degradation/destruction of the polymer will become the dominant process.

## 4. Conclusions

EVA coatings were successfully deposited by PED and PLD methods. The PED technique, which utilizes a pulsed electron beam to ablate the polymer, seems to be a more convenient way to produce good quality thin polymer films. This has been successfully shown for the EVA copolymer in the presented work. Nonetheless, the electron beam-macromolecule interactions are very complex and depend on the process conditions as well as on the targeted polymer structure, thus it is difficult to fully explain the mechanism of the PED process and further research is required. In the case of the EVA copolymer we believe that under electron ablation the VAc unit, which underwent the transformation to ketone functionality, became sufficiently structurally stable (as the C=O bond has a high bond dissociation energy) to maintain the general polymer structure and create a continuous thin film with some crosslinking state resulting from free radicals reactions. The quality of the EVA films produced using the PLD technique was much poorer in terms of chemical structure and film morphology. The main cause of this is believed to be the VAc block, which, besides heat and oxygen, is susceptible to UV light, undergoing strong photo- and thermo-degradation under photon radiation [4,24]. The worst results for the PLD coating deposition, in particular the structural changes, indicate that it is not such a universal polymer deposition technique as sometimes indicated in the literature [26], especially in the case of such complex materials as copolymers.

## Figures and Tables

**Table 1:** Elemental composition of the surface and quantitative deconvolution of C 1s spectrum.

Sample	Elements		C 1s Components with BE [eV]				
	C Total	O Total	C–C 285.0 eV	C*–CO 285.5 eV	C–OH C–O–C 286.3 eV	C=O 287.4 eV	O–C=O 289.2 eV
	at. %		C Total = 100				
EVA	89	11	59	22	11	3	5
EVA_E	82	18	53	21	9	12	5
EVA_L	82	18	49	22	12	9	8
